# Mortality Statistics in the Twelfth Census

**Published:** 1899-11

**Authors:** 


### MORTALITY STATISTICS IN THE TWELFTH CENSUS.

THE importance of securing accurate mortality statistics is well understood by all physicians and by most public men. One of the greatest difficulties in the way of obtaining accuracy is the want of uniformity in the system of mortality registration throughout the country. To obviate this the Hon. William R. Merriam, director of the twelfth census, has sent out a circular appealing to physicians and medical journals to coöperate with the census bureau in its effort to secure uniformity. The circular contains a form of death certificate, suggested by the statistician of the census, which we believe is worthy of adoption by all health officers in the United States. This improved form should be put into use for the calendar year 1900, which is the period selected for the collection of mortality returns.

The circular is a lengthy one, which should be obtained and read by all physicians; meanwhile, we present a synopsis of it prepared by the census office, as follows :

Physicians and students of mortality statistics will be interested in learning of the work now being accomplished by the chief statistician of vital statistics of the United States census, by the authority of the director, Hon. William R. Merriam. It is a practical effort, necessarily of limited scope, to secure the adoption of a uniform certificate for the return of deaths and looking toward the establishment of a common national system of collection of vital statistics for the purpose, primarily, of the census tables and publications.

Correspondence has been had by the chief statistician, Mr. William A. King, with the officers in charge of mortality registration in the states employing such a system, and in the cities having a population of 5,000 and more at the last census, which also collect and register death returns. Complete and accurate information of the different methods in vogue has been obtained, and it was found that there is much unnecessary and objectionable variation, considered from the census point of view, in the form of official returns.

Having no power to compel coöperative action and hampered by want of time in which to carry out the whole project, nevertheless the census office undertook to secure the modification or amplification of the death certificates so as to have them include the items necessary to obtain census data. A model return form was prepared and submitted, with explanatory correspondence, to each registration office or officer controlling the preparation of the state or local forms.

The result has been more important and gratifying than even the census office expected, as not only have the items in the specimen form been very generally adopted, but the registration officers have abolished many practically obsolete local variations in their certificates, and the latter have been made to conform to one standard more nearly than ever before.

The promptness and willingness displayed by the state and local officers in complying with the request of the director has been surprising as well as gratifying. The benefit that will result to the census office and to science from this first step toward the goal of national uniformity is incalculable, but it will be seen readily that the study of the natural law of the growth of the population is made easier and more certain.

The director of the census confidently expects that physicians everywhere will appreciate the desirability of the new order of things, and that they will earnestly and actively coöperate in securing prompt and accurate mortality returns of the uniform character required by congress and sought for by statisticians. He recognises the fact that failure on the part of physicians to give vitality to the common standard by carefully reporting the items that may be new to their certificate will be fatal to the end in view.

---

THE *Buffalo Commercial* in a recent issue thus calls attention to one of the entirely inexcusable annoyances of this city :

Is it absolutely necessary for public health and comfort that garbage shall be collected from private residences at five o'clock in the morning, and that the collectors should make a point of waking up every man, woman and child in the block ? Is there any mysterious, logical or inevitable connection between such an infernal racket, and public health and cleanliness?

This is a matter of which the JOURNAL has spoken repeatedly. It is a vexation to the well. It is a positive injury to the sick, to whom the sleep of the early morning hours is of almost vital importance. A little agitation in the daily press might result in doing away with one of the minor ills of city life. It takes a very long time for people to learn how much noise is unnecessary. Collectively the race is not so advanced as the individual and in the matter of city noises it is yet in its childhood.

---

AN INKLING of the backwardness of the Boers may be had from the newspaper accounts of the English campaign in the Transvaal, where in an official report it is stated that the British had captured a Boer hospital "equipped with the most primitive appliances."

---

THE ordinance requiring the muzzling of all dogs in the City of Buffalo went into effect on Wednesday, October 25, 1899. Violation of the ordinance is punishable by a fine of from $25 to $100.

This may have the effect of stamping out the epidemic of rabies, which seems to have made Buffalo its headquarters. A most interesting statement made in connection with this state of affairs is that the rabies originated in Ontario, Canada. We are of the opinion, however, that it is not possible to trace the source of the outbreak.

---

THE christian scientists of Buffalo are still holding out their prayer rugs and waiting for the aldermanic committee's report on the health commissioner's ordinance. The report will not be made until after the votes are counted and then the christian scientists will begin to report the cases of infectious and contagious diseases which they are praying over and getting money from.

